# Access to systemic anti-cancer therapies for women with secondary breast cancer—protocol for a mixed methods systematic review

**DOI:** 10.1186/s13643-021-01761-y

**Published:** 2021-07-23

**Authors:** Sally Anne Pearson, Sally Taylor, Antonia Marsden, Janelle Yorke

**Affiliations:** 1 Division of Nursing, Midwifery and Social Work, University of Manchester, Oxford Rd, Manchester, M13 9PL UK; 2 Christie Patient Centred Research, The Christie NHS Foundation Trust, 550 Wilmslow Road, Manchester, M20 4BX UK; 3 Division of Population Health, Health Services Research and Primary Care, University of Manchester, Oxford Rd, Manchester, M13 9PL UK

**Keywords:** Access, Systemic anti-cancer therapies, Secondary breast cancer, Inequity unwarranted variation

## Abstract

**Background:**

It is well recognised that access and receipt of appropriate guideline recommended treatment with systemic anti-cancer therapies for secondary breast cancer is a key determinant in overall survival. Where there is disparity in access this may result in unwarranted variation and disparity in outcomes. Individual, clinical and wider contextual factors have been associated with these disparities, however this remains poorly understood for women with secondary breast cancer. The purpose of the review is to examine individual, clinical and contextual factors which influence access to evidence-based systemic anti-cancer therapies for women with secondary breast cancer. This will include barriers and facilitators for access and receipt of treatment and an exploration of women and clinicians experience and perspectives on access.

**Methods:**

A mixed methods approach with a segregated design will be used to examine and explore factors which influence access to systemic anti-cancer therapies for women with secondary breast cancer. Electronic databases to be searched from January 2000 onwards will be EBSCO CINAHL Plus, Ovid MEDLINE, Ovid EMBASE, PsychINFO and the Cochrane Library and JBI database. This will include NHS Evidence which will be searched for unpublished studies and gray literature.

Title and abstract citations and full-text articles will be screened by the author and second reviewer. Data will be extracted by the author and validated by the second reviewer.

An overarching synthesis will be produced which brings together quantitative and qualitative findings. Methodological quality and risk of bias will be assessed using the Mixed Methods Appraisal Tool.

**Discussion:**

Understanding individual, clinical and wider contextual factors associated with access and receipt of systemic anti-cancer therapies for secondary breast cancer is a complex phenomenon. These will be examined to determine any association with access. Review findings will be used to guide future research in this area and the development of an evidence-based service level intervention designed to address unwarranted variation in access based upon the Medical Research Council (MRC) approach to the development, implementation and evaluation of complex interventions.

**Systematic review registration:**

The review protocol has been registered in PROSPERO CRD42020196490.

**Supplementary Information:**

The online version contains supplementary material available at 10.1186/s13643-021-01761-y.

## Background

Equity in access is a global health priority with adequate and fair access being fundamental to the organisation and delivery of high quality health services. Equity of access was considered to be one of the founding principles of the NHS with equitable access to treatment being essential to promote and preserve health [1]. Equity of access is a central component of the UK National Cancer Programme [2] however it is acknowledged that there remains inequity and unwarranted variation in access to treatment across the UK, which disproportionately influences health outcomes [3, 4]. With regard to cancer treatment it is accepted that access and receipt of appropriate therapy is a key determinant in improving outcomes. Conversely a number of factors have been identified as influencing access. However no attempt to date has been made to evaluate factors commonly associated with access to systemic anti-cancer therapies (SACT) for women with secondary breast cancer (SBC).

SBC has been defined as the development of new tumours in tissues and organs away from the primary tumour site [5] with the most common sites of metastases being lungs, liver, bones, and brain. It is estimated that up to 30% of women with primary breast cancer will experience disease recurrence following initial treatment and despite the increasing effectiveness of treatments, median overall survival is estimated at 3 years with no statistically significant improvement in the past twenty years [6]. SBC represents a significant disease burden with high levels of unmet need and unwarranted variation in outcomes [4]. Disparities have been reported across the breast cancer continuum which have compromised proper access to and receipt of services, however this remains poorly understood for women with SBC [7]. Guideline recommended SACT for the treatment of SBC is intended to optimize patient care informed by the best available evidence and an assessment of the benefits and harms of alternative treatment options. Treatment aims to improve long term survival, increase progression free survival and improve quality of life, though is seldom used with curative intent [8]. Current guidelines for SACT treatment for SBC are set out internationally, nationally and regionally and for the purposes of the review guideline recommended SACT are those which have been recommended by National, regional and local bodies.

From a theoretical perspective access has been defined as the “degree of fit between clients and the system” characterised by dimensions of availability, accessibility, accommodation, affordability and acceptability [9]. A more contemporary, patient centred framework where multi-level determinants related to health systems, institutions, organisations and providers are considered alongside factors at the individual, household, community, and population levels [10] will provide a theoretical basis for the review.

Review findings will be used to inform the development of an evidence-based service level intervention, based upon the UK Medical Research Council (MRC) approach to the development, implementation and evaluation of complex interventions [11]. The intervention will be designed to promote greater standardisation and a reduction in unwarranted variation in access and receipt of treatment. To achieve this, the review aims to identify the available evidence to investigate factors which influence access to guideline recommended treatment with systemic anti-cancer therapies for women with secondary breast cancer. This will include the identification of barriers and enabling factors and explore women and clinicians experience of access and treatment receipt for SBC.

## Methods

The protocol is reported in accordance with the PRISMA-P statement [12] (see Additional file 1) and has been registered with the International Prospective Register of Systematic Reviews (PROSPERO) registration number PROSPERO CRD42020196490.

### Aim, design and setting

The review will adopt a mixed methods approach applying a segregated design which will maintain the conventional binary distinction between the qualitative and quantitative paradigm, with integration of each distinct synthesis. The quantitative component aims to identify and investigate those factors associated with access and treatment. This will be complimented by the qualitative component which aims to explore how women with secondary breast cancer and their clinicians experience access and receipt of SACT (Table 1.). In particular to explore barriers and facilitators related to individual, clinical and wider contextual factors relating to health care systems and geographical location. A mixed method approach will be taken as access to treatment is a complex, multi-faceted phenomena which requires a comprehensive, patient centred understanding of how and why and in what context any association exists. This is conducive to a more sophisticated integration of the traditional quantitative and qualitative paradigms offered by a mixed methods approach.

**Table 1:** Inclusion and exclusion criteria

	**Study inclusion criteria**	**Study exclusion criteria**
Population:	Studies which report women aged > 18 and with a confirmed SBC diagnosis who accessed/received treatment with SACT. This will include studies which report all clinical sub types across all sites of metastases and will include de novo presentations	Studies which report primary and/or locally advanced (LABC) early stage (I-III) breast cancer only Studies which report (comparative) treatment effect and efficacy Males with a secondary breast cancer diagnosis as this is classified as a rare disease
Phenomena of interest:	Studies which explore individual, clinical and contextual factors associated with access to SACT for SBC	Studies which do not explore individual, clinical and contextual factors associated with the primary outcome
Intervention/Exposure:	Studies reporting individual factors, which include, age, gender, sexual orientation, race/ethnicity, socioeconomic status, education, language and literacy, psychosocial characteristics Clinical characteristics which include, sub type, HR status, HER2 status, previous treatment response, physician characteristics Contextual factors which include geography and geographical location, distance, travel time, health region, catchment/referral areas and organisational factors including, health care system factors, capacity, service availability	Studies which do not report individual, clinical and contextual factors associated with the primary outcome
Outcome(s):	Studies which report access in terms of receipt non receipt of one or more a systemic anti-cancer therapy(ies) For the purposes of the review access will be defined as receipt/non receipt of a systemic anti-cancer therapy which will include chemotherapy, immunotherapy, targeted therapy and hormone/endocrine therapy	Studies which do not report the primary outcome measure of receipt/non receipt of one or more a systemic anti-cancer therapy(ies)
Types of study:	Quantitative, qualitative and mixed methods studies. This will include observational, cross sectional, longitudinal and analytic studies, including, epidemiological studies, case control and cohort studies Qualitative studies will include designs such as phenomenology, grounded theory, ethnography, action research and feminist research Mixed method studies will only be considered if data from the quantitative or qualitative components can be clearly extracted Peer reviewed, original research studies published in English language from January 2000 onwards reporting quantitative, qualitative or mixed methods	Studies which report (comparative) treatment effect, efficacy and studies reporting clinical trials of systemic anti-cancer therapies, as whilst RCT is not a specific exclusion criteria it is likely that RCTs will measure treatment effect as opposed to access as an outcome and would therefore be excluded

The review setting and context reflects the mixed method approach and will consider studies which explore multi-level factors which have previously been associated with access to treatment. The review will consider studies which investigate and explore access to SACT, including chemotherapy, targeted therapy, immunotherapy and hormone/endocrine therapy. This will include studies which report across secondary, tertiary, specialist and palliative care centres, where SACT is accessed as either, first, second or subsequent line of treatment.

### Study eligibility

Criteria for study eligibility were developed to address the aims of the review and are set out in Table 1.

### Search strategy and information sources

A modified PICO framework was used to develop the review question [13]. This was then used to formulate the search strategy by identifying key concepts, subject headings and keywords (see example search strategy in Additional file 2). A preliminary search was undertaken to identify articles on the topic with text words in titles and abstracts of relevant articles. This was then used to develop a full search strategy. The search strategy including all identified keywords and index terms will be tailored to each information source. The electronic databases to be searched will be The Cochrane Library, EBSCO CINAHL Plus, Ovid MEDLINE, Ovid EMBASE, PsychINFO. Searches will be conducted from 1 January 2000 onwards. Reference lists of included studies will also be screened. The basic search strategy will be tailored to individual databases and undertaken with the support of an experienced librarian/evidence specialist. Grey literature will be searched using NHS Evidence.

### Study screening and selection

Records will be stored and managed in EndNote X9. Titles and abstracts will be screened against the inclusion and exclusion criteria. Potentially eligible studies will be retrieved in full. Full text articles will then be assessed against the inclusion and exclusion criteria, with reasons for exclusion recorded and reported. Screening and full text assessment will be undertaken by two independent reviewers with any disagreement resolved through discussion. Where consensus cannot be reached this will be achieved through arbitration with a third reviewer. The results of the search will be reported in full in the final report and presented in a Preferred Reporting Items for Systematic Reviews and Meta-analyses (PRISMA) flow diagram [14].

### Data extraction

Data will be extracted using an adaptation of a standardized data extraction tool which will be piloted prior to commencement of full data extraction. Data will be extracted for author, year of publication, study design, setting, country, primary data source and study population. Baseline population demographics will be extracted with primary exposure variables and covariates. Clinical characteristics will be extracted for diagnosis, clinical sub type of disease i.e. hormone receptor and HER2 status, performance status and comorbidities. Contextual factors for geographical location, population density and place of care will be extracted where reported. No data assumptions and simplifications are planned. Main findings will be extracted and assigned a level of credibility based upon methodological quality and risk of bias.

### Assessment of methodological quality and risk of bias

Methodological quality of included studies will be assessed using the Mixed Methods Appraisal Tool (MMAT) [15]. This will be undertaken by two independent reviewers with any disagreement resolved through discussion or where required with arbitration from a third reviewer. This process will be followed for quantitative and qualitative studies. The results of critical appraisal will be reported in narrative form accompanied by a summary table. Risk of bias for included studies will be classified as low risk, high risk, or unclear (either lack of information or uncertainty over the potential for bias). The Cochrane Collaboration tool for assessing risk of bias will be used for included randomised controlled trials and the preliminary risk of bias in non-randomised studies—of exposures (ROBINS-E) tool will be used for non-randomised interventional studies [16, 17]. Studies will not be excluded based upon low methodological quality or credibility, however this will be reflected in the analysis and synthesis.

### Data analysis, synthesis and integration

The review will follow a segregated approach to analysis and synthesis. This will involve distinct quantitative and qualitative analysis and synthesis followed by integration of the resultant evidence. Analysis and synthesis of quantitative included studies will be presented in narrative form together with tables and figures to aid data presentation. The narrative will be structured around multi-level factors which have shown an association with access and receipt of treatment as the primary outcome measure for the review. This will be determined a priori guided by the theoretical model of access adopted to guide the review [10]. Factors associated with access and receipt will be classified as individual, clinical and contextual. Where possible data will be pooled using statistical meta-analysis. This will be undertaken in accordance with guidance for meta-analysis of observational studies where appropriate. Heterogeneity will be assessed using the standard chi squared and I2 tests [18]. Analyses will be performed using a random effects model to estimate a summary measure of access and treatment receipt for each factor, with an overall summary measure of the likelihood of receipt of treatment for each included study. Qualitative findings will be presented in narrative form using a narrative synthesis approach [19]. Where possible these will be pooled using meta-aggregation to produce a comprehensive set of synthesized qualitative findings. Final synthesis of quantitative and qualitative findings will combine the separate synthesis into a set of conclusions which will reflect the narrative synthesis of qualitative findings and the quantitative findings produced from meta-analysis (where appropriate) to configure a mixed research synthesis.

## Discussion

Understanding multi-level factors associated with access and receipt of SACT for SBC is a complex phenomenon. Examining these factors in a comprehensive way will enable the development of evidence based strategies to address the challenges which they may present. It is acknowledged that appropriate access is a key determinant in overall survival, yet disparities have compromised proper access to and receipt of services resulting in unwarranted variation in outcomes. However, the extent of these disparities, the underlying reasons and the impact on outcomes remains poorly understood. Several theoretical frameworks have been proposed to conceptualise access. The multi-level factors and complexity of SACT treatment pathways for SBC led to the selection of a patient centred model of access to guide the review. The model was selected as it provided a contemporary perspective on access based on a synthesis of the published literature. The model incorporated dimensions of accessibility which represent supply, which when integrated with the corresponding abilities of persons to interact, generate access. The review will address those individual and clinical characteristics which influence the ability of individuals to interact with dimensions of accessibility to create access alongside dimensions of accessibility relating to wider contextual factors, including organisational and health care systems factors.

Practical issues in the conduct of the review may include identification of potentially eligible studies which may be compounded by the diversity of reported factors and characteristics within and between studies. There may be potential challenges in accurately and consistently identifying and quantifying multi-level factors which may be associated with access. Additionally the range of included study designs may present challenges in the assessment of methodological quality and subsequent analysis and synthesis. The review protocol has been designed to address these issues and minimise the impact in the conduct of the review. Conversely the complexity of the review question meant this could be most appropriately answered using a mixed methods approach, in particular a segregated design where qualitative and quantitative findings are viewed distinctly as complementary and synthesis is configurative.

Potential limitations may include high levels of heterogeneity between studies which may preclude statistical meta-analysis of included quantitative studies. There may be limited qualitative and mixed method studies identified in the topic area which would limit the potential for configurative synthesis and integration of quantitative and qualitative findings at review level. There may be potential challenges in accurately and consistently identifying and quantifying multi-level factors which may be associated with access. From a methodological perspective this will be addressed using narrative synthesis and where appropriate, sub group analysis for each factor to determine the extent and significance of the association. This will be further explored in the qualitative analysis and brought together through the synthesis. The inclusion of observational studies considerably expands the challenges in establishing a level of inference with a greater risk of confounding resulting from selection bias. Additionally, including only references published in English could lead to selection and reporting bias, which will be addressed through the analysis. Any amendments made to this protocol when conducting the review will be outlined in PROSPERO and reported in the final manuscript. In summary, the importance and significance of answering the review question outweighs the balance of the potential limitations. The impact of these have been reflected in the methodology and will be accounted for and addressed in the analysis, synthesis and integration of findings.

## Supplementary Information

**Additional file 1.** Updated PRISMA-P checklist.

**Additional file 2.** Example MEDLINE search strategy.

## Data Availability

Not applicable.

## Abbreviations

MRC: Medical Research Council
MMAT: Mixed Methods Appraisal Tool
SACT: Systemic Anti-Cancer Therapies
SBC: Secondary Breast Cancer
