# Supplementary material for: Access to systemic anti-cancer therapies for women with secondary breast cancer—protocol for a mixed methods systematic review
Source: Syst Rev. 2021 Jul 23;10:209. doi: 10.1186/s13643-021-01761-y (PMC8299679; doi:10.1186/s13643-021-01761-y)
Supplement: Supplementary file 2 — Additional file 2. Example MEDLINE search strategy. [file 13643_2021_1761_MOESM2_ESM.docx]

**Additional file 2. Example Medline search strategy:**

Database: Ovid MEDLINE(R)

Search Strategy:

--------------------------------------------------------------------------------

1 secondary.mp. (822053)

2 Neoplasm Metastasis/ or metastatic.mp. [mp=title, abstract, original title, name of substance word, subject heading word, floating sub-heading word, keyword heading word, organism supplementary concept word, protocol supplementary concept word, rare disease supplementary concept word, unique identifier, synonyms] (262196)

3 (oncology or cancer).mp. or Neoplasms/ (1738999)

4 (breast* or mammary).mp. (514766)

5 1 or 2 (1010611)

6 3 and 4 and 5 (52182)

7 sociodemographic.mp. [mp=title, abstract, original title, name of substance word, subject heading word, floating sub-heading word, keyword heading word, organism supplementary concept word, protocol supplementary concept word, rare disease supplementary concept word, unique identifier, synonyms] (40044)

8 socioeconomic.mp. [mp=title, abstract, original title, name of substance word, subject heading word, floating sub-heading word, keyword heading word, organism supplementary concept word, protocol supplementary concept word, rare disease supplementary concept word, unique identifier, synonyms] (208795)

9 ethnicity.mp. or Ethnic Groups/ (110127)

10 race.mp. or Continental Population Groups/ (109882)

11 psycho*.mp. [mp=title, abstract, original title, name of substance word, subject heading word, floating sub-heading word, keyword heading word, organism supplementary concept word, protocol supplementary concept word, rare disease supplementary concept word, unique identifier, synonyms] (1780208)

12 geograph*.mp. [mp=title, abstract, original title, name of substance word, subject heading word, floating sub-heading word, keyword heading word, organism supplementary concept word, protocol supplementary concept word, rare disease supplementary concept word, unique identifier, synonyms] (170506)

13 location.mp. [mp=title, abstract, original title, name of substance word, subject heading word, floating sub-heading word, keyword heading word, organism supplementary concept word, protocol supplementary concept word, rare disease supplementary concept word, unique identifier, synonyms] (257546)

14 distance.mp. [mp=title, abstract, original title, name of substance word, subject heading word, floating sub-heading word, keyword heading word, organism supplementary concept word, protocol supplementary concept word, rare disease supplementary concept word, unique identifier, synonyms] (187247)

15 7 or 8 or 9 or 10 or 11 or 12 or 13 or 14 (2600298)

16 access.mp. [mp=title, abstract, original title, name of substance word, subject heading word, floating sub-heading word, keyword heading word, organism supplementary concept word, protocol supplementary concept word, rare disease supplementary concept word, unique identifier, synonyms] (268620)

17 utili?ation.mp. [mp=title, abstract, original title, name of substance word, subject heading word, floating sub-heading word, keyword heading word, organism supplementary concept word, protocol supplementary concept word, rare disease supplementary concept word, unique identifier, synonyms] (205451)

18 health care.mp. or "Delivery of Health Care"/ (760344)

19 systemic.mp. (453643)

20 (anti cancer or anticancer or sact).mp. [mp=title, abstract, original title, name of substance word, subject heading word, floating sub-heading word, keyword heading word, organism supplementary concept word, protocol supplementary concept word, rare disease supplementary concept word, unique identifier, synonyms] (105641)

21 therap*.mp. [mp=title, abstract, original title, name of substance word, subject heading word, floating sub-heading word, keyword heading word, organism supplementary concept word, protocol supplementary concept word, rare disease supplementary concept word, unique identifier, synonyms] (5832457)

22 chemotherap*.mp. [mp=title, abstract, original title, name of substance word, subject heading word, floating sub-heading word, keyword heading word, organism supplementary concept word, protocol supplementary concept word, rare disease supplementary concept word, unique identifier, synonyms] (439040)

23 antineoplastic.mp. or Antineoplastic Agents/ (513050)

24 cancer drug.mp. [mp=title, abstract, original title, name of substance word, subject heading word, floating sub-heading word, keyword heading word, organism supplementary concept word, protocol supplementary concept word, rare disease supplementary concept word, unique identifier, synonyms] (6076)

25 drug therapy.mp. or Drug Therapy/ (2332816)

26 hormone therapy.mp. [mp=title, abstract, original title, name of substance word, subject heading word, floating sub-heading word, keyword heading word, organism supplementary concept word, protocol supplementary concept word, rare disease supplementary concept word, unique identifier, synonyms] (12826)

27 endocrine therapy.mp. [mp=title, abstract, original title, name of substance word, subject heading word, floating sub-heading word, keyword heading word, organism supplementary concept word, protocol supplementary concept word, rare disease supplementary concept word, unique identifier, synonyms] (6698)

28 tamoxifen.mp. or Tamoxifen/ (25980)

29 (oestrogen* or estrogen*).mp. [mp=title, abstract, original title, name of substance word, subject heading word, floating sub-heading word, keyword heading word, organism supplementary concept word, protocol supplementary concept word, rare disease supplementary concept word, unique identifier, synonyms] (191501)

30 epidermal growth factors.mp. or "EGF Family of Proteins"/ (967)

31 16 or 17 or 18 (1135295)

32 19 or 20 or 21 or 22 or 23 or 24 or 25 or 26 or 27 or 28 or 29 or 30 (6366026)

33 (biological therapy or Biological Therapy).mp. [mp=title, abstract, original title, name of substance word, subject heading word, floating sub-heading word, keyword heading word, organism supplementary concept word, protocol supplementary concept word, rare disease supplementary concept word, unique identifier, synonyms] (4334)

34 antibodies monoclonal.mp. or Antibodies, Monoclonal/ (215897)

35 Methotrexate/ (38211)

36 immunotherapy.mp. or Immunotherapy/ (93422)

37 32 or 33 or 34 or 35 or 36 (6499257)

38 6 and 15 and 31 and 37 (227)

***************************
